# Calibration to Mitigate Near-Field Antennas Effects for a MIMO Radar Imaging System †

**DOI:** 10.3390/s21020514

**Published:** 2021-01-13

**Authors:** Ha Hoang, Matthias John, Patrick McEvoy, Max J. Ammann

**Affiliations:** 1 Antenna & High Frequency Research Centre, Technological University Dublin, D08 NF82 Dublin, Ireland; matthias.john@tudublin.ie (M.J.); patrick.mcevoy@tudublin.ie (P.M.); max.ammann@tudublin.ie (M.J.A.); 2 Department of Telecommunications Engineering, Ho Chi Minh City University of Technology, Ho Chi Minh City, Vietnam

**Keywords:** near-field antenna effect, radar calibration, MIMO radar, turntable radar, UWB radar, radar system, scattering imaging, inverse scattering problem, radar resolution

## Abstract

A calibration method for a high-resolution hybrid MIMO turntable radar imaging system is presented. A line of small metal spheres is employed as a test pattern in the calibration process to measure the position shift caused by undesired antenna effects. The unwanted effects in the antenna near-field responses are analysed, modelled and significantly mitigated based on the symmetry and differences in the responses of the MIMO configuration.

## 1. Introduction

Turntable radar imaging systems for high-resolution imaging of complex objects can observe objects in arbitrary orientations and with a minimum of equipment [1]. No relative motion takes place between sensors and other objects in the environment, which can be utilised to eliminate environment effects in order to increase system accuracy. The long duration due to mechanical spatial-scanning is a disadvantage of a single-sensor or single-input single-output (SISO) turntable system. In a multiple-sensor or multiple-input multiple-output (MIMO) system [2,3,4,5,6,7], the sensors can be spread spatially to excite/observe the object in different spatial positions including position, direction and polarisation. In a high-speed capturing system, measurement data about the object can be captured with only one snapshot [8,9,10,11] with multiple spatial positions in the excitation/observation. Complexity and cost of the system are proportional to the number of spatial sensor positions.

A hybrid combination of a turntable and a minimalistic MIMO system is a suitable trade-off between increasing capture speed and reducing complexity and system cost. Moreover, differential features in the MIMO channel responses are very important and cannot be identified in a SISO system. These can be utilised for system calibrations to mitigate the system errors and/or system imperfections.

The calibration for a practical system plays a vital role in the improvement of reconstructed image accuracy [12]. The accuracy of nonideal electromagnetic acquisition systems is affected by many factors, in which near-field characteristics of antennas are a significant factor. The imperfection of $S_{11}$ characteristic of a bidirectional mode antenna and mutual near-field coupling between transmitting-receiving pairs of monodirectional mode antennas in the MIMO system are factors degrading the system accuracy. To eliminate these, the switching methods to turn off the receivers in the transmitting periods can be used, however this method is not suitable for a near-field radar system. Both the imperfection in $S_{11}$ and the mutual near-field coupling could be considered as an unknown mutual coupling of the system antennas. In [13,14], this effect in the MIMO systems was mitigated by a subtraction between the two measurements with and without the object. While, blind calibration processes were implemented in [15,16] assuming known antennas positions and direction from the objects. An experimental study of antenna array calibration [17] revealed a mismatch if only the coupling effect of antennas was considered. This can be explained by another undesirable effect in antenna responses that is magnitude, latency/phase and polarisation in transmitting/receiving responses of the antennas dependent on direction. In [13], an adaptive weighting technique was proposed to calibrate this directional dependence error based on the measurement data at the exact positions. To tackle both the unknown mutual coupling and the antenna directional-dependence, the reflected signals from the metal plane in different positions were measured and processed in the calibration in [18,19]. Besides using the passive/static objects in calibration, the active/reconfigurable objects were also used in calibration processes as a beacon in [20] or a rotatable double-antenna polarimetric active radar calibrator in [21]. However, the measurement data at the exact spatial positions was also a requirement of these methods.

In this article, a hybrid MIMO turntable radar imaging system [12] and a calibration method to reduce the undesirable effects of the antennas on the system performance are reported. The undesired antennas effects in the MIMO system configuration are analysed in a perspective of near-field propagation [22]. Additionally, the effects of the object rotation using the system turntable are analysed and the impacts on the estimation of object position is modelled, investigated and measured. The calibration scheme is proposed to mitigate these effects in order to improve system accuracy with a minimisation of the complexity in measurement arrangement.

## 2. Radar Imaging System

**Figure 1:**
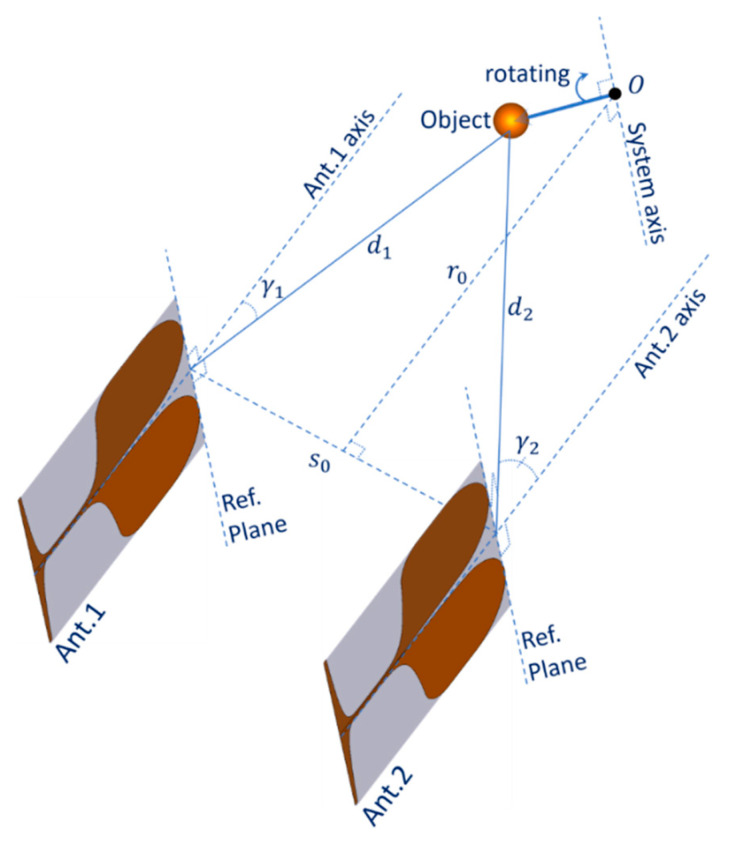
System configuration.

Figure 1 shows the configuration of the MIMO turntable radar imaging system [12]. It includes two vertical fixed-mounted Vivaldi antennas and a turntable facilitating the rotation of measured objects around the system axis with an angular step size Δβ. The two antennas Ant.1 and Ant.2 are parallel, with a distance of $s_{0}$ between them and have the same distance to the system axis. The two antennas axes are in the system plane. The system axis is perpendicular to the system plane and cuts this plane at the system origin O. The distance from the system axis to the plane containing the two antennas reference planes is $r_{0}$. The two antennas are connected with two bidirectional ports of a Rohde & Schwarz ZVA 40 vector network analyser (VNA) playing the role of a frequency-sweeping transceiver of the radar system. The system parameters are shown in Table 1.

Spatial tolerances in system configuration and object arrangements impact the accuracy of the imaging system. Reducing the spatial tolerances is challenging and increases the cost in setting up the system as well as arrangement of the objects. However, the calibration scheme proposed in the Section 4 can reduce the effect of some of these spatial tolerances.

In this MIMO system, each antenna plays the role of a transmitting antenna, a receiving antenna or both and time division multiplexing (TDM) of the VNA is used to divide each measurement period into the two time slots. In each time slot, concurrently, one antenna is in transmitting (Tx) and receiving (Rx) mode while the other is in Rx mode. The modes of the antennas alternate in the next time slot.

With two antennas and the turntable, there are four combinations for spatial observing channels (corresponding to four active radar virtual observing angles to the objects space) in the measurement of object scattering characteristics at each position of the mechanical rotation. These channels are presented in Table 2. The mechanical rotation of the turntable includes 240 steps with $1.5^{{}^{\circ}}/\mathrm{step}$. Thus, the total number of (virtual) observing angles to the objects space can reach to 4×240.

In practice, the speed of electrical mode switching for the antennas is faster than mechanical state change for the turntable. Thus, this hybrid MIMO configuration is able to increase the density of (virtual) observing directions to the objects space and/or increase data acquirement speed when compared to a turntable single-channel configuration. The time-domain inverse scattering algorithm [23,24,25] is applied to reconstruct the object scattering image from radar measurement data. The object scattering images can be produced based on measured data of all of mechanical rotation angles and corresponding to each mechanical rotation angle, data of one or all of four channels are used for this reconstruction.

The inverse scattering algorithm is based on the propagating waves in the system model to identify scattering sources. The accurate modelling for the real propagation process on/between the antennas and the objects is necessary [22]. However, in this algorithm, each antenna is considered as working in the far-field relative to the objects, isolated from other antennas and with ideal $S_{11}$, so the effects of the real operational antenna conditions significantly degrade the system accuracy. These antenna effects are addressed, modelled and mitigated in the next sections. Additionally, the rotation of the objects in the measurement process and the reconstruction algorithm is also concerned and investigated in terms of its effect on estimated object-position in the next section.

## 3. Antennas Effects and Shift Modelling

**Figure 2:**
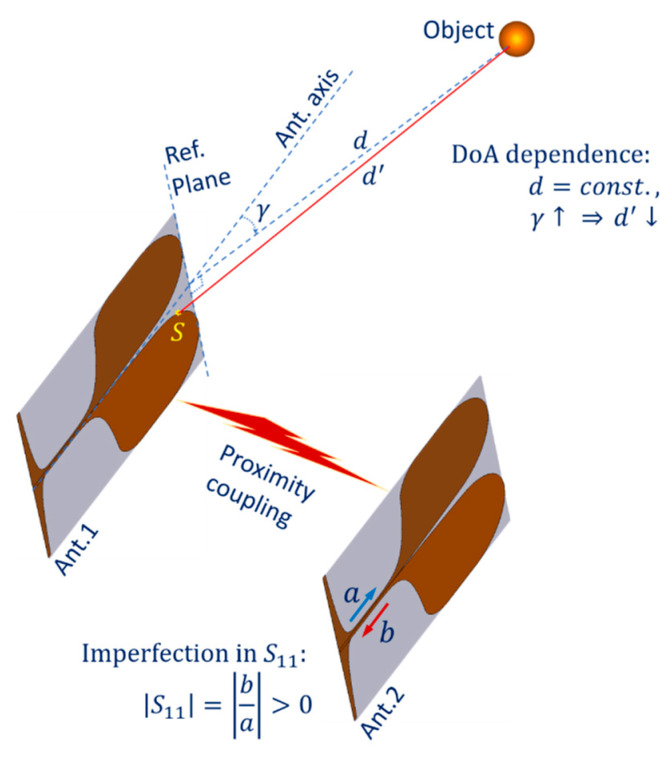
Undesired antennas effects.

In the case of an antenna operating concurrently in both transmitting or receiving mode (C11 or C22), the proportion of electromagnetic (EM) energy reflecting/scattering back to the antenna port at discontinuities in the structure (e.g., at the end edge, lateral edge or at the connector) is the main factor causing imperfection in $S_{11}$ antenna characteristic [22]. This effect can occur many times between parts of the structure, forming higher-order reflection components visible in the received signal in time domain. Both first-order and higher-order reflection components of the dominant transmitted signal can mask the small amount of received EM energy scattered from the target object and is received by the antenna. The imperfection in $S_{11}$ is illustrated in Figure 2 by the fact that $\left|S_{11}\right|$ parameter is always greater than zero in practice.

Another undesired effect in the system configuration is the proximity coupling between the two antennas. In the case of the observing channel being C12 or C21, this can be considered as a mutual coupling channel between the transmitting and receiving antennas of distance $s_{0}$. The first-order scattering components from the transmitting antenna can be received by the receiving antenna. In the case of the observing channel being C11 or C22, mutual coupling also occurs, the inactive antenna appears as a distributed target in close proximity to the active antenna. Only second- and higher-order scattering components from the inactive antenna can be received by the active antenna. Thus, in the case of C11 or C22, the received signal is affected by the both $S_{11}$ imperfection and mutual coupling effects. In all cases C11, C22, C12 or C21, the coupling EM energy proportion received at the receiving antenna can mask the desired signal scattered from the object. The proximity coupling between the two antennas of the system is also illustrated in Figure 2.

**Figure 3:**
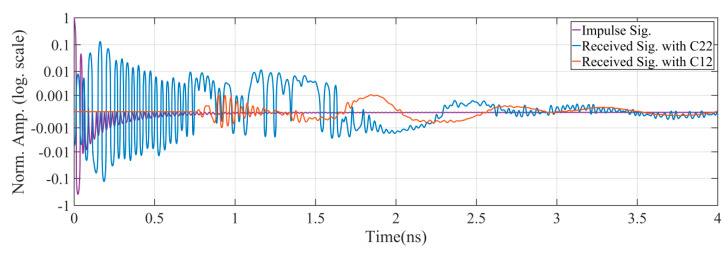
Received signals measured at the antenna port with the effects of imperfection in $S_{11}$ and proximity coupling.

Figure 3 shows the impulse signal and the measured received signals at the antenna ports. It is observed that the intrinsic antenna structure causes significant reflections, even from the regions of the connection port as the reflecting signal is formed from the beginning of the impulse. The amplitude of this reflection signal (Received Sig. with C22) is significantly greater than the amplitude of received signal caused by the proximity coupling (Received Sig. with C12). Due to the distance $s_{0}$ between the two antennas, there is a corresponding latency in the proximity coupling signal (Received Sig. with C12). Another observation is that the higher-order scattering components of the two antennas lead to the elongation of both received signals in the time domain.

Another undesired effect of the Vivaldi antennas is dependence on direction of arrival (DoA). An example of a ray-model propagation path for part of the scattered EM energy propagating from the object to the antenna port is shown in Figure 2. When the antenna 1 (Ant.1) acts as a receiver, this path starts from the object, propagates over the subpath in the air $d^{\prime }$ to the scattering point S on the Vivaldi edge and propagates over the rest of the Vivaldi edge toward the antenna port. The direction of the path is reversed when the antenna is in transmitting mode. Assuming that the distance d from the reference plane of the antenna to the object is not changed, when the arrival angle γ increases, while the far-field model shows that d is constant versus γ, the subpath in the air $d^{\prime }$ of the ray-model decreases. This decrease phenomenon also happens to any arbitrary ray from the object to any point on the antenna element behind the referent plane. Thus, the practical equivalent length of the subpath in the air by multipath superposition of all rays scattering from the object to the antenna patches also has a corresponding decline versus γ. This DoA dependence effect leads to a significant error when the inverse scattering algorithm is applied to reconstruct the scattering image of the object space if only the far-field model is used.

**Figure 4:**
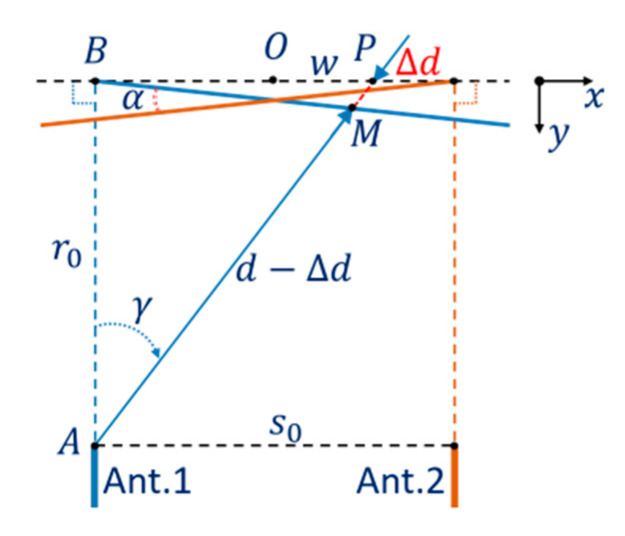
Shift model of antenna DoA dependence effect.

The effect of DoA dependence of the Vivaldi antennas to a shift in object position in the measurement result is explained in Figure 4. When Ant.1 measures an object at P, the position in the measurement result is shifted to M by Δd, which is a function of the angle γ. For objects at points in the segment $\left[-s_{0}/2\;\;\;s_{0}/2\right]$ of the x axis around the system origin O, the dependence of the shift Δd on γ can be approximated by a linear function versus tan(γ) or the distance BP, this is shown by the blue line (BM) in Figure 4. Symmetrically, the function of the shift when measured by Ant.2 is represented by the orange line and the angle between the two lines is α. This angle α can be considered as a differential characteristic parameter of the DoA dependence effect of the two antennas in the MIMO system.

The relationship between the shift Δd and the system parameters, the differential parameter α, and the observing angle γ from Ant.1 to the object can be formulated based on the trigonometric relation of the edge lengths and the vertex angle in the triangle ABP:

(1) $$\tan\left(\gamma \right)=\frac{\left(\frac{s_{0}}{2}+w\right)}{r_{0}}.$$

When combined with the relationship in the triangle BPM and the projections of Δd on the x axis—Δd sin(γ) and on the y axis—Δdcos(γ):

(2) $$\tan\left(\frac{\alpha }{2}\right)=\frac{\Delta d\cos\left(\gamma \right)}{r_{0}\tan\left(\gamma \right)-\Delta d\sin\left(\gamma \right)}.$$

Thus, the equation for the shift Δd versus γ and the system parameters can be written:

(3) $$\Delta d=\frac{r_{0}\;\tan\left(\gamma \right)\tan\left(\frac{\alpha }{2}\right)}{\cos\left(\gamma \right)+\sin\left(\gamma \right)\tan\left(\frac{\alpha }{2}\right)}.$$

**Figure 5:**
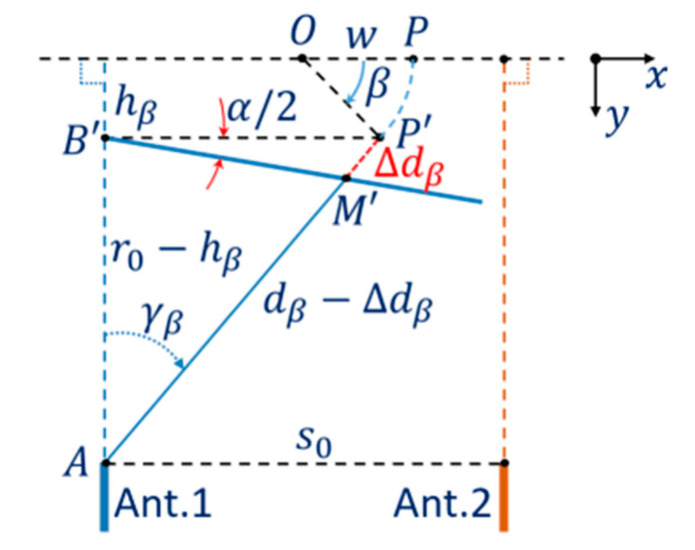
Rotation effect on the shift model of antenna DoA dependence effect.

In this work, the estimation of the position of the object is based on measuring the distance to the object rotating around the system axis and applying the inverse scattering algorithm to the measurement data set in order to reconstruct the object image. Thereby, the shift caused by the antenna DoA dependence effect can be evaluated. However, effects of the rotation in the measurement can affect significantly the estimation results. Figure 5 shows the shift model with the rotation effect. In each step of the measurement process by Ant.1, when the turntable rotates by an angle β from the initial position, the object at P is moved to P′ and the measurement result for the position of P′ is moved to M′ with the shift $\Delta d_{\beta }$ caused by the antenna DoA dependence effect. This shift can be considered as a function of the variable β.

Assuming that the differential parameter α does not depend on the distance from the antenna to the measured position or the distance AB′. The relationship between the shift $\Delta d_{\beta }$ and the parameters and rotating variable β can be formulated as follows.

The projections of the segment OP′ on the x and y directions are:

(4) $$w_{\beta }=w\;\cos\left(\beta \right),\;\;h_{\beta }=w\;\sin\left(\beta \right).$$

In the triangle AB′P′,

(5) $$\frac{s_{0}}{2}+w_{\beta }=\frac{s_{0}}{2}+w\;\cos\left(\beta \right),$$

(6) $$d_{\beta }=\frac{r_{0}-h_{\beta }}{\cos\left(\gamma_{\beta }\right)}=\frac{r_{0}-w\;\sin\left(\beta \right)}{\cos\left(\gamma_{\beta }\right)}$$

and

(7) $$\tan\left(\gamma_{\beta }\right)=\frac{\frac{s_{0}}{2}+w\;\cos\left(\beta \right)}{r_{0}-w\;\sin\left(\beta \right)}$$

or

(8) $$\gamma_{\beta }=\mathrm{artan}\left\{\frac{\frac{s_{0}}{2}+w\;\cos\left(\beta \right)}{r_{0}-w\;\sin\left(\beta \right)}\right\}.$$

Considering the trigonometric relationship in the triangle B’P’M’, the projections of $\Delta d_{\beta }$ on the x and y directions and the Equation (7) then

(9) $$\tan\left(\frac{\alpha }{2}\right)=\frac{\Delta d_{\beta }\cos\left(\gamma_{\beta }\right)}{\left\{r_{0}-w\;\sin\left(\beta \right)\right\}\tan\left(\gamma_{\beta }\right)-\Delta d_{\beta }\sin\left(\gamma_{\beta }\right)}.$$

Thus, the shift $\Delta d_{\beta }$ can be formulated as a function of the variable β, the object position and the system parameters:

(10) $$\Delta d_{\beta }=\frac{\left\{\frac{s_{0}}{2}+w\cos\left(\beta \right)\right\}\tan\left(\frac{\alpha }{2}\right)}{\cos\left(\mathrm{artan}\left\{\frac{\frac{s_{0}}{2}+w\cos\left(\beta \right)}{r_{0}-w\sin\left(\beta \right)}\right\}\right)+\sin\left(\mathrm{artan}\left\{\frac{\frac{s_{0}}{2}+w\cos\left(\beta \right)}{r_{0}-w\sin\left(\beta \right)}\right\}\right)\tan\left(\frac{\alpha }{2}\right)}.$$

**Figure 6:**
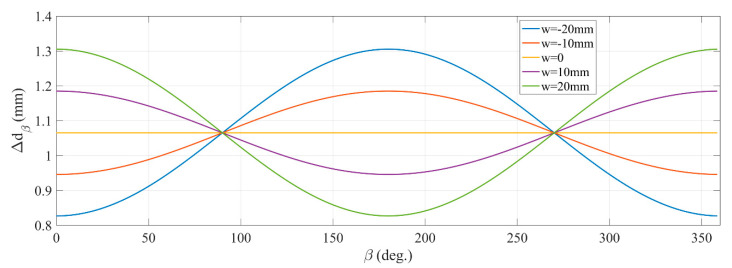
An investigation of rotation effect to the shift.

An investigation of the shift $\Delta d_{\beta }$ versus angle β with different initial x-axis object positions is implemented. With w=0, the objects initial position is at the origin O, with w<0 on the left and with w>0 on the right of the origin. This investigation is implemented with the differential parameter α = 1.35°. The plots in Figure 6 show that the shift $\Delta d_{\beta }$ of each point varies with the rotation angle β depending on distance w from the origin to the initial position. Another feature is that at β=0 the average of $\Delta d_{\beta }$ is greater than the initial value (Δd without the rotation effect) for points on the left of the origin and less for points on the right. Thus, if the estimation of Δd is based on averaging over β, then the estimated value of Δd tends to increase on the left of the origin and to decrease on the right. This demonstrates that the estimated function of Δd is nonlinear versus tan(γ) or distance BP.

Considering the superposition in the reverse scattering algorithm to the response signals scattered from the objects with rotation, local peaks in the reconstructed image tend to spread out and shift with measured spatial errors $\Delta d_{\beta }$. However, because of nonlinear or non-sawtooth shape around peaks of the time-domain response signals corresponding to the measuring frequency band, this superposition leads to shrinking of the spatial errors in mapping to the reconstructed image. Thus, the value of the shift Δd estimated from the reconstructed image tends to be smaller than its value in the model. The above analyses show that there are differences between the shift model in Equation (3) and the measured and estimated result of the shift with the effects of the rotation and the feature of the inverse scattering reconstruction algorithm.

An adjusted model for the shift with effects of the rotation and the feature of the reconstruction algorithm is proposed. Considering the feasibility for the measurement and estimation of the shift caused by the DoA dependence of the antennas in this work, the adjusted model for the shift Δd is still a proportional function versus tan(γ) as Equation (3), but the differential parameter α is replaced by $\alpha_{a}$ with a k factor, 0<k<1 as per Equation (11). The model is used in a calibration scheme for the system in next section.

(11) $$\alpha_{a}=k\alpha$$

## 4. Calibration Scheme and Results

To mitigate the effects of the imperfection of $S_{11}$ characteristic and mutual proximity coupling between the two antennas, the background subtraction method [23,25] is applied. However, the slow ripple over environment temperature in the response of the system transceiver can reduce the effectiveness of this method. The distance $r_{0}$ from the antenna reference planes to the system x axis is chosen large enough that undesired scattering components described above arrive earlier than the scattered signals from the object. Thereby, the error of higher-order components at the object-scattering period, caused by the slow ripple in the transceiver, is small enough compared to the intensity of the scattered signals from the object. Limiting the range of the angle γ considering the width of the target object is also a factor in the choice of the lower bound of $r_{0}$. The upper bound of $r_{0}$ depends on the intensity of scattering signals from the objects compared to the system noise level. In this work, $r_{0}$ is chosen as 690 mm.

Spatial tolerance in the alignment and positioning of the system components and calibration objects significantly affects the system accuracy. In the system, the fixed connected components such as the two antennas can be aligned accurately together with little additional effort. However, high-accuracy alignment and positioning for the separate parts of the system can require a lot of effort and high-cost measurement equipment.

**Figure 7:**
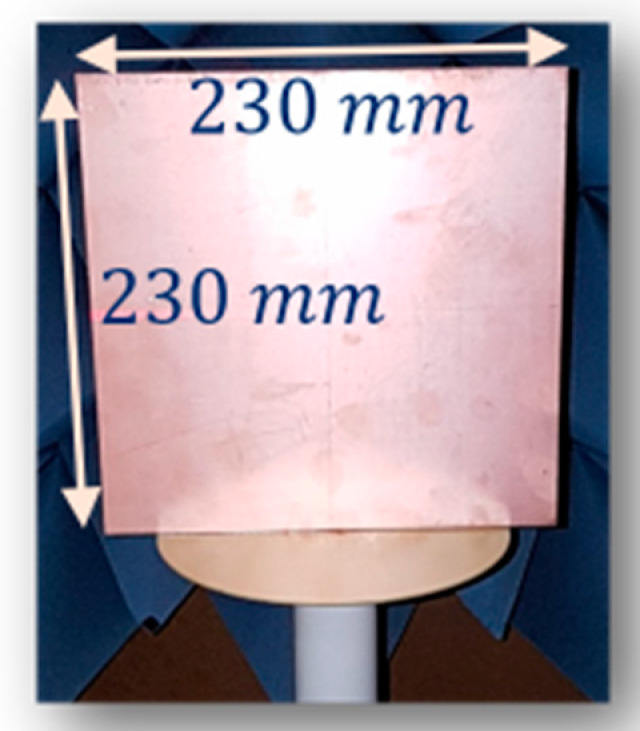
Reflector aligned at rotating axis of the turntable for the system origin calibration.

To simplify alignment and positioning for the system origin, a calibration for the nominal distance $r_{0}$ of the system is implemented based on an equivalent distance calculated from the measured propagation time (from the antennas to the system origin). This measurement is implemented with a planar reflector placed at the system origin. To reduce the rigour in alignment of the reflecting plane in the measurement, the highly directional characteristic in radar cross-section of the reflecting plane and the high-resolution in rotation of the system turntable are utilised. The plane is rotated to find the balance in azimuth angle of the plane to the two antennas corresponding to the peak of the received signal with the observing channel C12 or C21, in which the propagation path starts from one antenna, propagates to the plane and reflects to the other antenna. The propagation time of the path corresponding to the case of the balance in azimuth angle of the plane is used to estimate the equivalent distance from the antennas to the system origin and correct the $r_{0}$ parameter. Figure 7 shows the $230\times 230\;{\mathrm{mm}}^{2}$ reflecting plane aligned on the rotating axis of the turntable used in this calibration.

**Figure 8:**
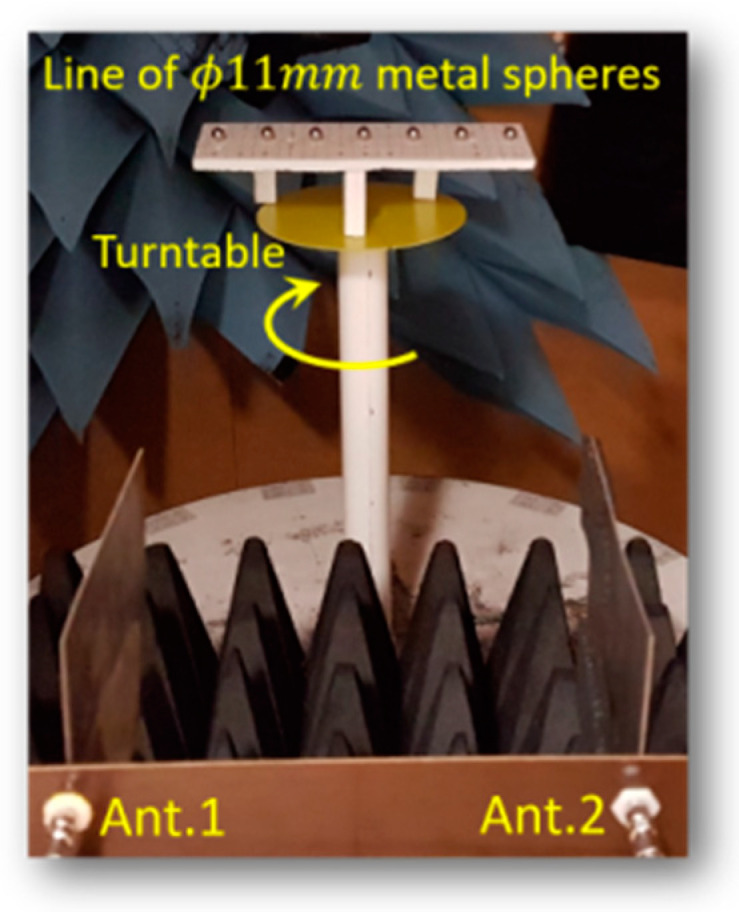
Measurement of the differential parameter by a line of metal spheres.

As mentioned in Section 3, the antenna DoA dependence effect causes a shift in the estimated object position when the estimation is based on reconstructed images. This shift in the MIMO system was modelled and characterised by the differential parameter $\alpha_{a}$ as presented in the Equations (3) and (11). The measurement for the differential parameter $\alpha_{a}$ uses a calibration pattern of seven steel spheres of 11 mm diameter evenly spaced 40 mm apart and aligned close to the horizontal line at the system origin. Figure 8 shows the pattern and its location in the system. Radar measured data is acquired based on the received signals of both antennas operating in monostatic mode (observing channel C11 and C22) at each rotating step of the turntable.

**Figure 9:**
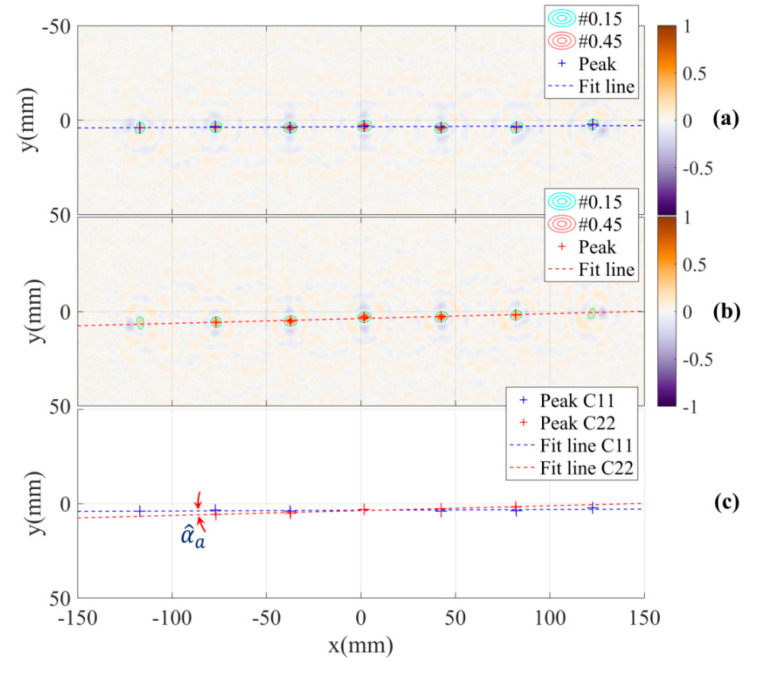
Reconstructed images with the DoA dependence effect based on measurement channels (**a**) C11, (**b**) C22 and (**c**) the estimation of differential parameter of the shift.

Figure 9a,b shows the reconstructed images of the calibration pattern based on two sets of radar data measured by channels C11 and C22 with the DoA dependence effect. The first observation from the images is that the peak values corresponding to object positions far from the system origin (image centre) are smaller than the values close to the origin. This can be explained by the rotation effect on the superposition of the reconstruction algorithm, there is a proportional increase in the fluctuation in the shift of object positions farther from the system origin, this leads to an increase in the spread of image energy around these peak positions and degrades these peak values.

The shift phenomenon caused by the antenna DoA dependence effect can be evaluated based on these two images. Firstly, the positions of the objects in the two images are estimated using a local peak finding algorithm. Next, in each image, a line across the objects positions is estimated by fitting a linear function with the peak positions set. The angle of the two lines from the two images represents the differential parameter $\alpha_{a}$ of the shift in the adjusted model. In this experiment, the estimated angle of the parameter $\alpha_{a}$ is ${\hat{\alpha }}_{a}=1.16^{{}^{\circ}}$. The peak positions, fitted lines and the angle between the two lines are shown in Figure 9c.

Due to the symmetry of the MIMO system and the use of differential angle of the two lines for the estimation, the evaluation of the shift parameter is not sensitive to tolerances in direction and space between the line of the calibration pattern and the system horizontal line. These tolerances can be caused by inaccurate alignment of the calibration pattern on the turntable. Additionally, by fitting a linear function on the peaks corresponding to the sphere positions, this function is a characteristic line of the peaks set with relative distance errors to the peaks. This suggests that in the alignment of the calibration pattern, straightness of the line of spheres is not a rigorous requirement.

**Figure 10:**
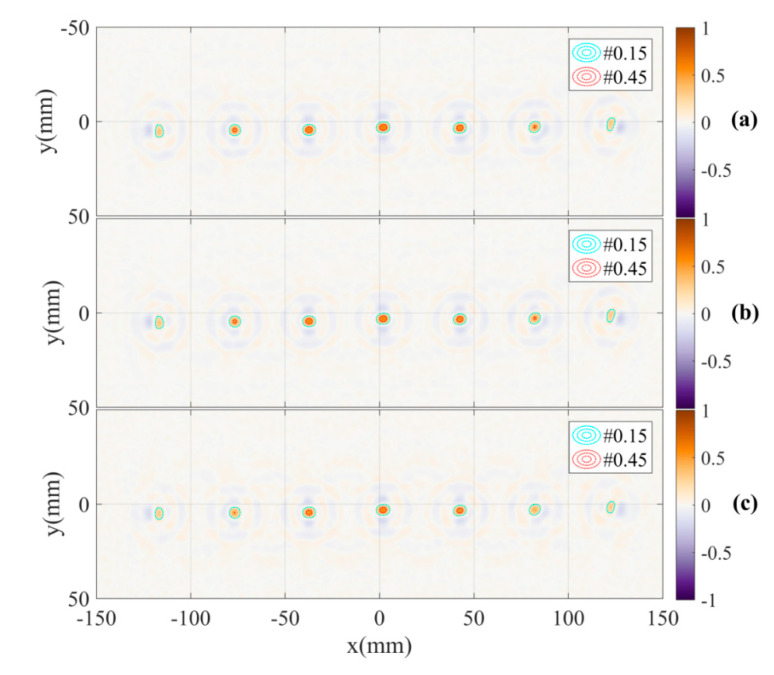
Reconstructed images with DoA dependence effect based on measurement channels (**a**) C12, (**b**) C21 and (**c**) all four channels.

For a comparison, the images of the calibration pattern reconstructed from radar data collected by channels C12, C21 and all of four channels are also presented in Figure 10.

Considering Equation (3) and the adjusted model of the shift with the effects of the rotation and the inverse scattering algorithm of the system, the estimated shift $\Delta \hat{d}$ for Δd in the model can be calculated from the estimated differential parameter ${\hat{\alpha }}_{a}$ by the equation:

(12) $$\Delta \hat{d}=\frac{r_{0}\;\tan\left(\gamma \right)\tan\left(\frac{{\hat{\alpha }}_{a}}{2}\right)}{\cos\left(\gamma \right)+\sin\left(\gamma \right)\tan\left(\frac{{\hat{\alpha }}_{a}}{2}\right)}.$$

**Figure 11:**
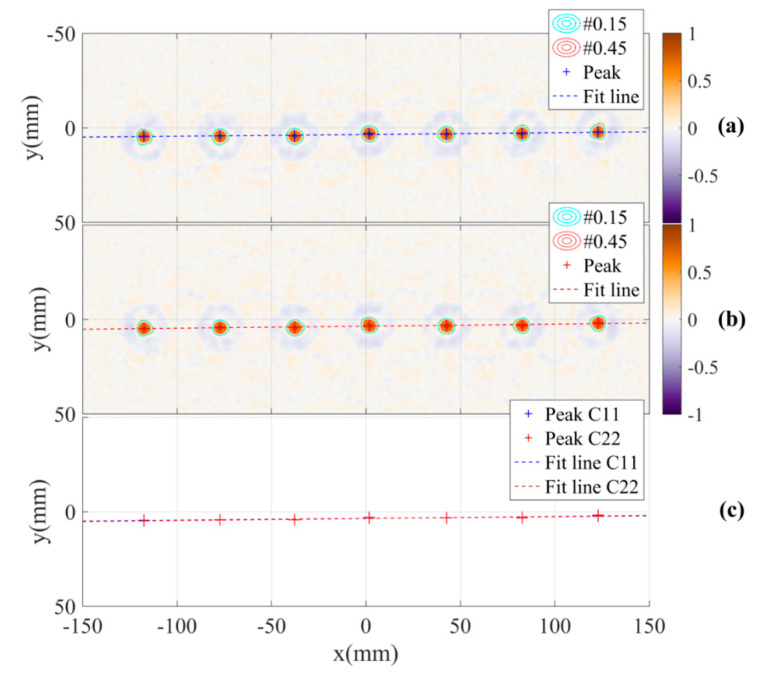
Reconstructed images with DoA dependence calibration based on measurement channels (**a**) C11, (**b**) C22 and (**c**) estimation of angle between the two lines.

**Figure 12:**
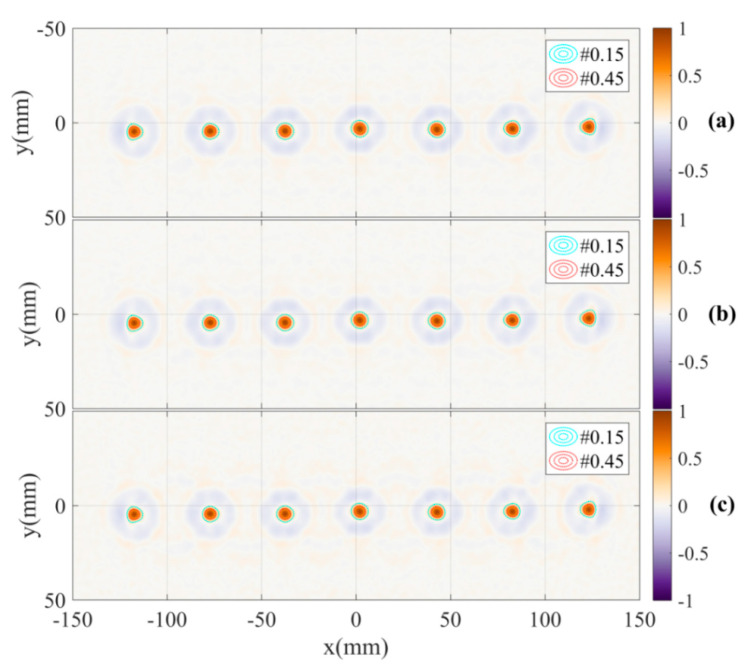
Reconstructed images with DoA dependence calibration based on measurement channels (**a**) C12, (**b**) C21 and (**c**) all four channels.

To mitigate the antennas DoA dependence effect, the shifts at each position in the object space corresponding to the distance of the paths from the object to the two antennas are compensated by the estimated shifts calculated by Equation (12) in the inverse scattering algorithm. The results of applying the compensation to the calibration pattern are shown in Figure 11. The images show the improvement in focus specifically the significant increase and regularity of the peaks. The antennas DoA dependence effect is significantly mitigated. This is demonstrated by the overlap of the peak positions of the two images and the reduction of the estimated angle between the two lines down to approximately $0^{{}^{\circ}}$ in the reconstructed images measured by channels C11 and C22. Additionally, the images of the calibration pattern reconstructed from radar data collected by channels C12, C21 and all of the four channels with DoA dependence calibration are also presented in Figure 12. These figures also show the effectiveness of the calibration.

**Figure 13:**
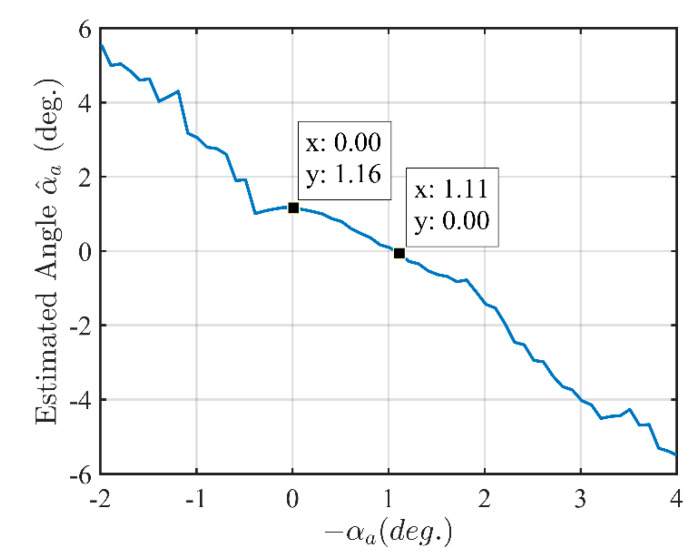
Angle between the two lines estimated from the reconstructed images vs. the angle $-\alpha_{a}$ used to compensate for the differential parameter $\alpha_{a}$ of the model.

An investigation of relationship between the angle $-\alpha_{a}$ used to compensate for the differential parameter $\alpha_{a}$ in the adjusted model and the angle between the two lines practically estimated from the reconstructed images is implemented based on the calibration pattern measured radar data. This investigates how the compensation affects the practical estimation of the angle between the two lines and whether a multisolution in the algorithm to eliminate the DoA dependence effect exists. The result in Figure 13 shows that if there is no compensation ($-\alpha_{a}=0$) then the estimated angle between the two lines is $1.16^{{}^{\circ}}$. The angle between the two lines is suppressed when the compensation is implemented by a value of $-\alpha_{a}=1.11^{{}^{\circ}}$. This also shows that the error between the differential parameter $\alpha_{a}$ of the model and the measured and estimated differential parameter ${\hat{\alpha }}_{a}$ is approximately $0.05^{{}^{\circ}}$. In this investigation with the range of $6^{{}^{\circ}}$ ($-2^{{}^{\circ}}$ to $4^{{}^{\circ}}$, step of $0.1^{{}^{\circ}}$) of the parameter $-\alpha_{a}$, there is only one solution for elimination of the antenna DoA dependence effect corresponding to $-\alpha_{a}=1.11^{{}^{\circ}}$. Therefore, the calibration algorithm has a univalent convergence in the range of the parameter $-\alpha_{a}$.

**Figure 14:**
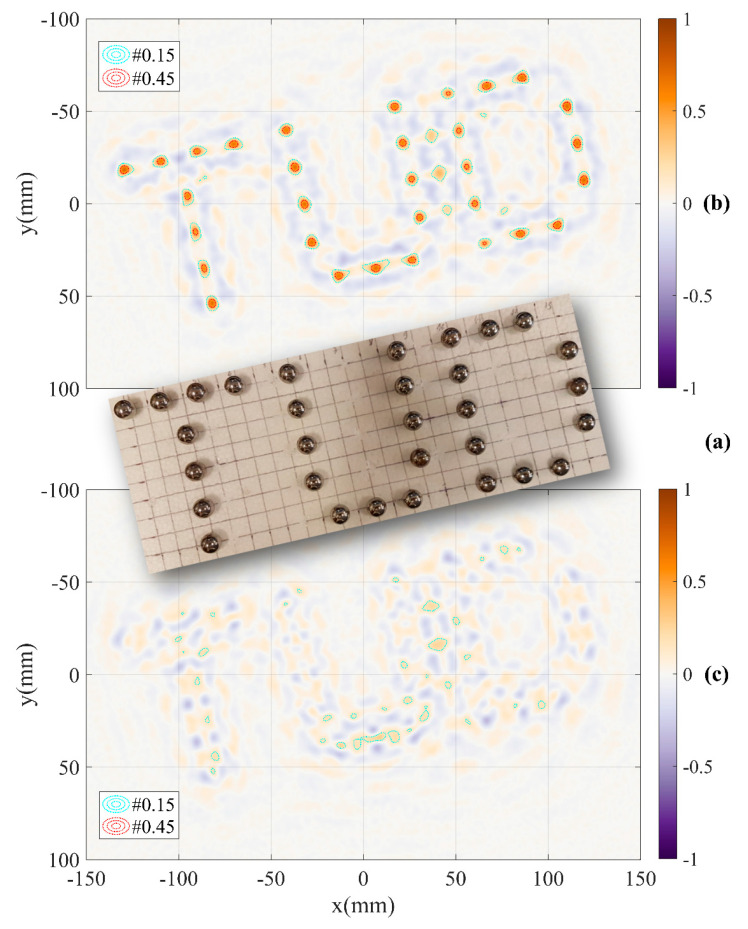
Testing the system with (**a**) “TUD” pattern and results (**b**) with DoA dependence calibration and (**c**) without DoA dependence calibration based on measurement data of all of four channels.

When the calibration parameter has been determined, the system can be used to measure other objects such as a pattern with 31 steel spheres of 11 mm diameter arranged into a shape of “TUD” characters. The distance between two adjacent centres of spheres is 20 mm. The pattern and reconstructed images with and without DoA dependence calibration are shown in Figure 14. The results show that with DoA dependence calibration, intensities of the peaks are high and moderately regular. The positions of the spheres in the “TUD” pattern can be identified accurately based on these peaks as seen in Figure 14b. While without DoA dependence calibration, the peaks or the positions of the objects cannot be identified as shown in Figure 14c. This comparison demonstrates a significant effectiveness of the calibration method.

However, the quality of the reconstructed image in Figure 14b is lower than that of Figure 12c. We observe the appearance of phantom peaks (e.g., between the “U” and “D” characters) and a reduced and irregular intensity of object peaks. This is caused by the increase in number of spheres (from 7 to 31), the decrease in distance between the objects (40 mm down to 20 mm) and the distribution of the objects in two dimensions of the system plane in the “TUD” pattern, when compared to the calibration pattern. These differences lead to more complexity in the propagation progress [22] between objects and antennas at each observed angle. The increased probability of an object being occluded by others is the main factor in the quality reduction of the reconstructed image.

## 5. Conclusions

This article presented a hybrid MIMO radar imaging system associated with the undesired near-field antennas effects and demonstrated the effectiveness of a calibration method to mitigate these effects. The calibration scheme was based on the analysis and modelling of the propagation process and differential features of the MIMO system configuration as well as tolerated the errors in the measurement arrangement. The advantage of the method was demonstrated in improved focus of image energy at object peaks in the reconstructed scattering images. This facilitates highly accurate near-field detection of small objects using antennas which are large compared to the object size.

## Figures and Tables

**Table 1:** System parameters.

Parameters	Values
Antennas space ($s_{0}$)	180 mm
System distance ($r_{0}$)	690 mm
Angular step size (Δβ)	$1.5^{{}^{\circ}}$
Rotating step number	240
Turntable tolerance	$\pm 0.1^{{}^{\circ}}$
Antenna size (L × W)	$130\times 120{\;\mathrm{mm}}^{2}$
System bandwidth	10 MHz–39.9 GHz
Frequency step	10 MHz
Transmitting power	10 dBm

**Table 2:** Spatial observing channels vs. antenna modes.

Channel	Antenna Mode
C11	Tx: Ant.1, Rx: Ant.1
C12	Tx: Ant.1, Rx: Ant.2
C21	Tx: Ant.2, Rx: Ant.1
C22	Tx: Ant.2, Rx: Ant.2
